# Autophagy in Age-Related Macular Degeneration: A Regulatory Mechanism of Oxidative Stress

**DOI:** 10.1155/2020/2896036

**Published:** 2020-08-08

**Authors:** Zi-Yuan Zhang, Xiao-Li Bao, Yun-Yi Cong, Bin Fan, Guang-Yu Li

**Affiliations:** Department of Ophthalmology, The Second Hospital of Jilin University, Changchun 130000, China

## Abstract

Age-related macular degeneration (AMD) is a leading cause of severe visual loss and irreversible blindness in the elderly population worldwide. Retinal pigment epithelial (RPE) cells are the major site of pathological alterations in AMD. They are responsible for the phagocytosis of shed photoreceptor outer segments (POSs) and clearance of cellular waste under physiological conditions. Age-related, cumulative oxidative stimuli contribute to the pathogenesis of AMD. Excessive oxidative stress induces RPE cell degeneration and incomplete digestion of POSs, leading to the continuous accumulation of cellular waste (such as lipofuscin). Autophagy is a major system of degradation of damaged or unnecessary proteins. However, degenerative RPE cells in AMD patients cannot perform autophagy sufficiently to resist oxidative damage. Increasing evidence supports the idea that enhancing the autophagic process can properly alleviate oxidative injury in AMD and protect RPE and photoreceptor cells from degeneration and death, although overactivated autophagy may lead to cell death at early stages of retinal degenerative diseases. The crosstalk among the NFE2L2, PGC-1, p62, AMPK, and PI3K/Akt/mTOR pathways may play a crucial role in improving disturbed autophagy and mitigating the progression of AMD. In this review, we discuss how autophagy prevents oxidative damage in AMD, summarize potential neuroprotective strategies for therapeutic interventions, and provide an overview of these neuroprotective mechanisms.

## 1. Age-Related Macular Degeneration

Age-related macular degeneration (AMD) is a leading cause of irreversible blindness in the elderly population [1] and is becoming a global crisis, with the number of affected people expected to reach 288 million by 2040 worldwide [2]. AMD is classified into two typical forms in the clinic, i.e., dry and wet, both of which can result in visual loss [3]. The wet form, also called exudative or neovascular AMD, is characterized by choroidal neovascularization (CNV) [4] with an abnormally increased expression of vascular endothelial growth factor (VEGF) [5]. The CNVs can leak fluid or blood into the subretinal space (SRS) and lead to sudden vision loss. In contrast, visual loss is usually gradual in the dry form [6]. Yellow subretinal deposits called drusen, or extracellular protein aggregates of retinal pigment epithelial (RPE) cells [7], as well as the accumulation of intracellular lipofuscin [8], can be found under an ophthalmoscope. Larger drusen may become confluent and evolve into drusenoid RPE detachments [9], which often progress to geographic atrophy and less frequently to neovascular AMD. Geographic atrophy is the main pathological feature of dry AMD and can lead to severe visual loss when involving the center of the macula [10].

Many factors determine the risk of developing AMD, including both genetic and environmental factors [11, 12]. Among them, oxidative stress [13–15] and senescence [16] are two major risk factors for AMD, and a growing body of evidence suggests that inflammation also plays an important role in the pathophysiology of AMD [17–19]. Senescence induced by chronic oxidative stress can inhibit cell growth and lead to the release of growth factors, cytokines, chemokines, proteases, and other molecules, inducing inflammation [20]. Additionally, a number of lifestyle factors, including smoking [21], improper dietary intake [22, 23], obesity [24], and lack of exercise [25], are associated with a higher prevalence of AMD. Cigarette smoke can cause accumulation of cadmium (Cd) [26] and further increase the oxidant load in retinal tissues [27]. Dietary zinc deficiency can sensitize RPE cells to oxidative damage [28]. A high-fat diet with excessive cholesterol may contribute to AMD, as the oxidized form of cholesterol, 7-ketocholesterol, is found at high levels in drusen [29]. The interactions among these factors remain elusive.

RPE cells play a critical role in the pathogenesis of AMD [30]. They are highly specialized pigmented cells located between the neuroretina and the choroid [31]. The physiological functions of RPE cells are essential to maintain the normal health of the retina [32]. These functions include phagocytosis of shed photoreceptor outer segments (POSs) [33], metabolism in the SRS [34], the formation of the outer blood-retinal barrier [35], the exchange of 11-cis retinol and all-trans-retinol during the retinoid cycle [36], and the regulation of ion and metabolite transport [37]. Alterations to retinal metabolism have been reported to be an early feature of AMD [2]. In the pathogenesis of AMD, age-related, cumulative oxidative stress can cause functional abnormalities of RPE cells and induce incomplete digestion of POSs, leading to the continuous accumulation of cellular waste [38]. The major cellular waste is drusen and lipofuscin (a metabolite in lysosomes), containing unfolded and damaged proteins [39] or DNA [40]. Under physiological conditions, these unnecessary proteins are cleared and recycled in RPE cells by two main systems of protein degradation: the ubiquitin-proteasome system (UPS) and autophagy [41]. However, overloaded cellular waste cannot be degraded completely by autophagy or UPS due to the progressive dysfunction of RPE cells in AMD. This will finally lead to cellular degeneration and subsequent death of photoreceptors because RPE cells lose the ability to provide them with oxygen and nutrients and remove waste materials [42].

## 2. The Role of Oxidative Stress in AMD

Oxidative stress is a major cause of AMD [43]. An imbalance between oxidation and antioxidation is induced when organisms are exposed to biotic and abiotic stress factors such as hypoxia [44]. The main characteristic of oxidative stress is the increased levels of reactive oxygen species (ROS), leading to morphological damage and functional weakness of cellular proteins, lipids, and DNA [45]. ROS include a variety of chemical substances, such as singlet oxygen, superoxide anion radicals, hydroxyl radicals, hydrogen peroxide (H_2_O_2_), and hydroxyl peroxide radicals [46]. ROS are generated during metabolic processes related to life-sustaining or enzyme-catalyzed reactions [46]. The retina is metabolically very active, maintaining normal physiological function, and thus, it consumes high amounts of oxygen and produces many ROS [47]. These ROS under physiologic conditions are conducive to signal transduction in the retina [48].

RPE cells are responsible for the phagocytosis of POSs as discussed before. POSs contain a lot of unsaturated fatty acids. In the process of POS phagocytosis, nicotinamide adenine dinucleotide phosphate oxidase or peroxidase in the phagocytic bodies will oxidize these fatty acids in POSs and generate large amounts of ROS [49]. Under stress conditions, photoreceptor cells have to metabolize constantly to renew their outer segments, which contribute to a unique source of ROS for RPE cells [50]. Moreover, RPE and photoreceptor cells contain higher levels of mitochondria, which are likely to produce more ROS than other cells [48]. However, unfavorable oxidative stress is triggered when ROS overaccumulate, causing disorders of the cell structure and function, which in turn aggravates ROS production [12]. Photooxidative stress is induced by light and is one of the forms of oxidative stress [51]. Studies have found that photooxidative stress can induce accumulation of deposits in RPE cells and eventually lead to the degeneration of RPE and photoreceptor cells [52]. Additionally, photosensitive molecules (rhodopsin and lipofuscin) interact with light; they are related to oxidative stress induction and the death of photoreceptor cells. Taken together, high oxygen metabolism, continuous light exposure, high concentrations of polyunsaturated fatty acids, and the existence of photosensitizers make the retina prone to be affected by oxidative stress [53].

**Figure 1:**
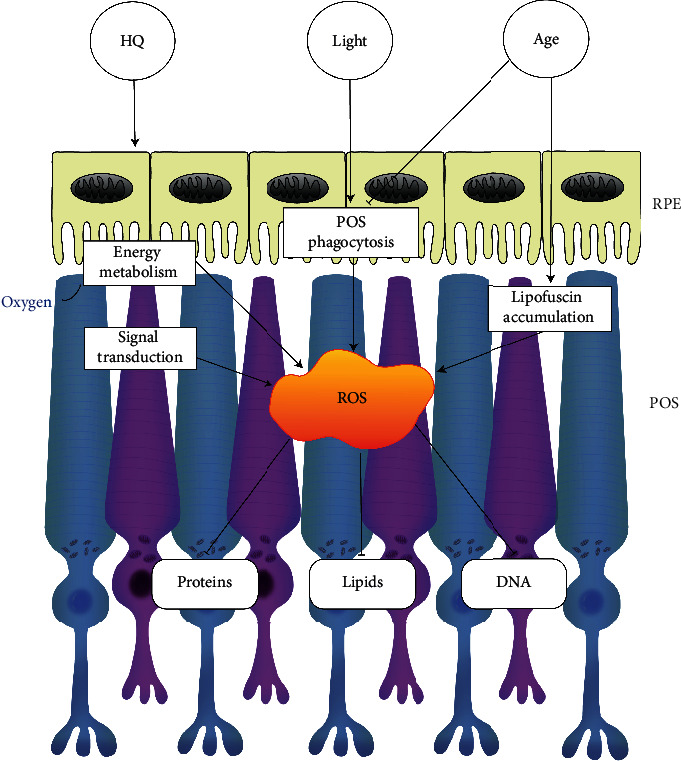
The role of oxidative stress in AMD. Light injury, growing age, and oxidants from cigarette smoke (such as HQ) are the risk factors for AMD. Overactive energy metabolism and excessive signal transduction in RPE and photoreceptor cells produce many ROS. Daily phagocytosis of POSs in RPE cells is also an important source of ROS. RPE cells lose the ability of phagocytizing POSs with increasing age, leading to the accumulation of lipid substances, such as lysosomal deposits (also known as lipofuscin). Taken together, ROS are elevated upon exposure to risk factors, and thus, cellular oxidative stress is triggered, causing injuries to proteins, lipids, and DNA and finally the death of RPE and photoreceptor cells. AMD: age-related macular degeneration; HQ: hydroquinone; RPE: retinal pigment epithelial; POSs: photoreceptor outer segments; ROS: reactive oxygen species.

Aging is related to progressive oxidative stress in the pathology of AMD [54]. With advancing age, the deposition of lipids and proteins in Bruch's membrane and RPE cells has a negative impact on physiological cell functions [55], resulting in reduced cell adhesion, proliferation, and migration and impaired POS phagocytosis. Lipofuscin is a kind of residue from poor lysosomal POS degradation [56]. In AMD, lipofuscin accumulation is induced due to dysfunction of degenerative RPE cells. The lipofuscin promotes oxidative stress by producing free radicals and inhibiting degradation of damaged organelles and proteins [57]. The relationship between lipofuscin and protein degradation systems will be discussed below. The overview of the role of oxidative stress in AMD is presented in Figure 1.

The main methods of inducing oxidative stress in AMD are increasing oxidative stimuli or dysregulating the antioxidant mechanisms [43]. Here, we introduce several common models of establishing AMD mediated by increased oxidative stress. Photooxidative stress models, also called light injury models, are widely used in the investigation of AMD [58]. One of the mechanisms of retinal injury is the interaction between light and photosensitive molecules. The excessive activation of rhodopsin and light conduction can induce photoreceptor cell degeneration [53]. H_2_O_2_, a component of ROS, is also widely used to stimulate oxidative stress in both in vivo animal models and in vitro RPE cell culture models [59]. Additionally, cigarette smoke, containing powerful chemical oxidants such as hydroquinone (HQ), Cd, and nicotine, can induce oxidative stress and disturb the proteasome pathway in cultured human RPE cells [60]. Furthermore, an autophagy deficiency model is considered to be a potential AMD model. Insufficient autophagy leads to the accumulation of lipofuscin and ROS. Interestingly, ROS and oxidized lipoproteins are also major causes of disturbed autophagy clearance [61]. Liu and colleagues showed that intravitreal injection of wortmannin, an autophagy inhibitor that can irreversibly block phosphatidylinositol 3-kinase (PI3K), transiently suppressed autophagy in C57BL/6J mice within a week, leading to RPE and photoreceptor cell degeneration and death [62].

## 3. The Role of Autophagy in AMD

Autophagy, which literally means “self-eating,” is a lysosome-dependent multistep process that is widely existent in eukaryotic cells. It can be divided into macroautophagy, microautophagy and chaperone-mediated autophagy. Macroautophagy, which can be either selective or nonselective, is the most studied and is considered to be the major autophagy pathway [63, 64]. In the process of macroautophagy, damaged organelles and protein aggregates are engulfed in a double-membrane vacuole to form autophagosomes, which are then transported to lysosomes to form autolysosomes for final degradation [65]. Recent studies have found that RPE cells are the major site of pathological alterations in AMD, and autophagy dysfunction in RPE cells plays a key role in the development of AMD [66]. The levels of autophagic flux in RPE cells from AMD donors have been found to be decreased compared with RPE cells from healthy controls [67]. These facts illustrate that autophagy is highly correlated with AMD. In this part, we mainly focus on the role of autophagy in the pathogenesis of AMD.

### 3.1. Autophagy and Lipofuscin

Lipofuscin, which is composed of covalently crosslinked proteins, lipids, and saccharides, is formed in RPE cells when lipoproteins accumulate due to the disturbed degradation of POSs and extracellular materials [41]. Autophagy is widely considered as a major protein degradation system. With increasing age, lipofuscin accumulates in RPE cells together with its primary spontaneous fluorophore, A2E, and contributes to the pathogenesis of AMD [8, 38]. Once formed, lipofuscin is hard to degrade; it exerts a toxic effect on RPE cells, for example, causing increased DNA damage, inhibiting proteolysis, and reducing cell viability in a time- and concentration-dependent manner [68]. Zhang and colleagues coincubated RPE cells with A2E and found that A2E could induce autophagy in RPE cells at an early stage [69]. It has also been shown that inhibiting autophagy could increase the levels of lipofuscin-like autofluorescence (LLAF), whereas enhancing autophagy by glucosamine targeting the 5′-adenosine monophosphate-activated protein kinase (AMPK)/mammalian target of rapamycin (mTOR) pathway could at least partially attenuate LLAF in RPE cells [38, 70]. These findings suggest that elevated levels of autophagy in RPE cells can abate the accumulation of lipofuscin, thereby preventing the adverse effects of A2E in RPE cells and potentially delaying AMD progression.

### 3.2. The Protective Role of Autophagy in AMD

In addition to age-related oxidative stress, high levels of oxygen consumption, exposure to lipid peroxidation products, and oxidative damage all make RPE cells susceptible to chronic oxidative stress [71]. RPE cells from AMD patients produce more ROS than normal RPE cells and lose the ability to increase superoxide dismutase (SOD) expression when exposed to continuous oxidative stress [72, 73]. Beclin-1 can regulate and induce autophagy [74]. Microtubule-associated protein 1 light chain 3 (LC3) has been considered to be a primary biochemical marker for autophagy activation. The conversion of the soluble form of LC3 (LC3-I) to the autophagic vesicle-associated form (LC3-II) is indicative of autophagic flux [75]. These are regarded as reliable autophagy markers. Several studies have shown that promoting autophagy through various signaling pathways, such as the PI3K/protein kinase B (Akt)/mTOR pathway [76, 77], the AMPK/mTOR pathway [38], the p62/Kelch-like-ECH-associated protein 1 (Keap1)/nuclear factor erythroid 2-related factor 2 (NFE2L2) pathway, and the peroxisome proliferator-activated receptor gamma coactivator 1 (PGC-1) pathway [78, 79], could reduce the occurrence of AMD. The levels of Beclin-1 and the ratio of LC3-II to LC3-I (LC3-II/LC-I) were found to be increased in these studies. Moreover, the NFE2L2 and PGC-1 pathways are also antioxidant pathways, suggesting that autophagy may perform important functions in the regulation of oxidative stress, which will be discussed below.

### 3.3. The Role of Mitophagy in AMD

Recently, the role of selective autophagy in AMD has been revealed. Double-membrane structures carrying specific cellular components interact with phagophores with the help of selective autophagy receptors and trigger selective autophagy [63]. These components include cytoplasmic aggregates (triggering aggrephagy), lipid droplets (triggering lipophagy), exogenous pathogens (triggering xenophagy), and organelles such as mitochondria (triggering mitophagy) [80]. Mitophagy is essential for maintaining proper cellular functions, since it participates in mitochondrial quality control and clears mitochondria with mutated mitochondrial DNA (mtDNA) [81]. It has been reported that aged RPE cells are more susceptible to oxidative stress, in which the efficacy of mitophagy decreases and mtDNA damage accumulates [82–84]. Hyttinen and colleagues showed that the number of mitochondria in RPE cells from AMD donors was lower than in those from healthy donors, and eight times more mtDNA damage than nuclear DNA damage was observed, indicating that mitophagy has a significant impact on the development of AMD [71]. Interestingly, NAD^+^, a critical component that can accelerate the metabolic shift towards glycolysis can also induce mitophagy, restoring homeostasis in RPE cells in AMD patients, and may therefore serve in novel AMD treatment strategies [2].

### 3.4. The Dual Role of Autophagy in AMD

Improving autophagy can mitigate the degeneration of RPE cells; however, an increasing number of studies have illustrated that excessive autophagy may also lead to retinal cell death [85], particularly overactivated autophagy at early stages of retinal diseases [86]. Zhang and colleagues showed that blocking autophagy directly or inhibiting autophagy by suppressing the mitogen-activated protein kinase (MAPK)/extracellular signal-related kinase (ERK) pathway could protect photoreceptor cells against light-induced damage [87]. Li and colleagues found similar results. They investigated the protective role of epigallocatechin-3-gallate (EGCG), a polyphenolic compound from green tea that protects against ultraviolet light-induced oxidative stress [88]. Surprisingly, although EGCG lowered ultraviolet light damage in an autophagy-dependent manner, it decreased the levels of LC3-II and the formation of autophagosomes instead of increasing them. This notable finding reminds us that autophagy may play a dual role in the protection against retinal degenerative diseases. Appropriate enhancement of autophagy can be beneficial, but excessive autophagy can be harmful and inhibit protective effects.

## 4. Autophagy Can Regulate Oxidative Stress in AMD

Recently, autophagy has been observed as a crucial regulatory mechanism of oxidative stress in AMD. As mentioned above, autophagy is one of the two major protein degradation systems and is essential to maintain homeostasis in RPE and photoreceptor cells. In AMD, the endocytic/phagosome and autophagy pathways are disturbed in degenerative RPE cells due to impaired cargo handling and processing. Keeling and colleagues proposed that this may contribute to increased proteolytic and oxidative stress, which results in irreversible injury to postmitotic RPE cells [89]. Moreover, autophagy has been found to be enhanced in response to oxidative stress, in order to remove oxidatively damaged proteins and organelles, since RPE cells are exposed to constant oxidative stress during the development of AMD [90].

Mitter and colleagues studied the role of autophagy under oxidative stress by culturing human ARPE-19 cells and exposing them to H_2_O_2_. They established models of both acute and chronic AMD, exposing cells to H_2_O_2_ for 6 hours and 14 days, respectively. Interestingly, they found that there was a dynamic alteration of autophagic flux in RPE cells exposed to oxidative stress: acute oxidative stress stimulated autophagic activity, whereas chronic oxidative stress resulted in a reduction of autophagic activity. Inhibition of autophagy by 3-methyladenine or by knockdown of *ATG7* or *BECN1* could increase lipofuscin accumulation and ROS generation. Lipofuscin is believed to inhibit autophagy by blocking the function of lysosomal enzymes and causing excess permeabilization of lysosomal membranes, which can lead to the release of lysosomal content and the subsequent production of more toxic radicals [91]. In contrast, oxidative stress-induced ROS production decreased after treatment with rapamycin to upregulate autophagy. Their findings demonstrate that autophagy is crucial to the resistance to oxidative stress in RPE cells, and defective autophagy is likely to exacerbate oxidative stress in AMD [92].

Chen and colleagues further confirmed the regulatory role of autophagy in photooxidative stress by using an *Abca4^–/–^ Rdh8^–/–^* mouse model. Photoisomerization of the visual chromophore 11-cis retinol and all-trans-retinol is an essential step of retinal photoelectric conversion to maintain normal vision. However, excessive production of all-trans-retinol may cause retinal cell death. *Abca4^–/–^ Rdh8^–/–^* mice are deficient in ATP binding cassette transporter 4 (ABCA4) and retinol dehydrogenase 8 (RDH8). These are crucial enzymes for all-trans-retinol clearance from photoreceptors. Thus, *Abca4^–/–^ Rdh8^–/–^* mice can develop light-dependent retinal degeneration due to delayed clearance of all-trans-retinol. The team showed that the protein levels of the autophagosome marker LC3-II and the mitophagy regulator Park2 were increased in *Abca4^–/–^ Rdh8^–/–^* mice upon light exposure. They also employed a Beclin-1-deficient mouse model and a rod photoreceptor-specific Atg7-deficient mouse model to inhibit autophagy, and they used *Park2^–/–^* mice to block mitophagy. These mice all exhibited severe retinal degeneration due to inadequate autophagy or mitophagy. Taken together, both autophagy and mitophagy perform critical functions in regulating photooxidative stress [93].

In summary, accumulating researches support that enhancing autophagic activity can alleviate oxidative stress in AMD and protect RPE and photoreceptor cells from progressive degenerations. However, it is still unknown whether autophagy plays a dual role in regulating oxidative stress. Below, we will illustrate some underlying mechanisms of autophagy regulating oxidative stress.

## 5. The Mechanisms of Autophagy Regulating Oxidative Stress in AMD

**Figure 2:**
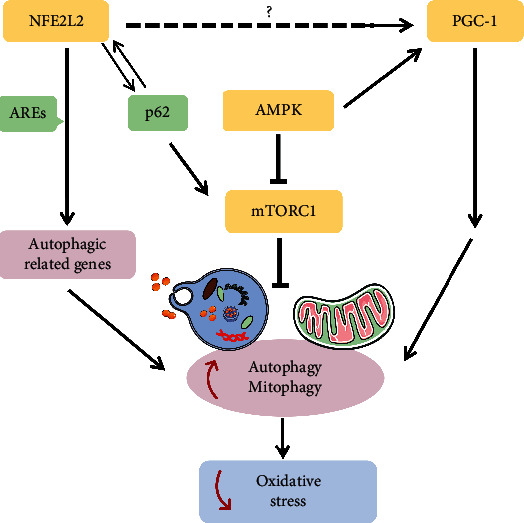
The interactions among the pathways involved in reducing oxidative stress by enhancing autophagy. NFE2L2 seems to be a positive regulator of PGC-1, but the specific functional mechanisms remain to be studied. NFE2L2: nuclear factor erythroid 2-related factor 2; AREs: antioxidant response elements; PGC-1: peroxisome proliferator-activated receptor gamma coactivator 1; AMPK: 5′-adenosine monophosphate-activated protein kinase; mTORC1: mammalian target of rapamycin complex 1.

Oxidative stress and autophagy can be therapeutic targets for AMD treatment. Recent studies have investigated the elusive link between autophagy and oxidative stress. Results indicate that autophagy plays an important role in alleviating oxidative stress and reducing retinal cell death. However, the specific mechanisms and signal pathways by which autophagy regulates oxidative stress remain to be studied. We have found that several mechanisms and pathways enhance autophagic activity to protect RPE and photoreceptor cells from oxidative stress. Here, we discuss the interactions among these pathways to explore how autophagy regulates oxidative stress in AMD. The overview of the interactions among these pathways is presented in Figure 2.

### 5.1. Interactions among NFE2L2, p62/SQSTM1, and mTOR Pathways

NFE2L2 signaling has been found to be a critical pathway that mediates autophagy and oxidative stress [66]. NFE2L2, also known as Nrf2, is a transcription factor that has protective effects against ROS-induced retinal cell death. NFE2L2 can bind to antioxidant response elements (AREs), activate the expression of nuclear and metabolic genes, and regulate DNA replication, transcription, mitochondrial function, and cell growth [94], protecting cells from oxidative damage. Inactive NFE2L2 can bind to the cytoskeletal protein Keap1 and then stays in the cytosol [95]. Upon oxidative stress, cytosolic NFE2L2 is phosphorylated and translocated to the nucleus in response to protein kinase C activation and MAPK pathways. In the nucleus, NFE2L2 activates proteasomal subunits and the expression of autophagy-related genes through AREs [96] by interacting with transcription factors in the bZip family, including CREB, ATF4, and FOS or JUN. Gene activation through NFE2L2 can be blocked by small Maf proteins, such as MafG and MafK, to balance NFE2L2 action and regulate the intracellular oxidation levels [97].

The scaffolding adaptor protein p62, also known as sequestosome 1 (SQSTM1), can be selectively cleared by autophagy [98]. Phosphorylated p62/SQSTM1 can bind to LC3 or ubiquitin, promoting the degradation of unnecessary protein aggregates and malfunctioning mitochondria by autophagy [99]. Thus, the amount of p62/SQSTM1 is inversely proportional to the autophagic flux. In addition to autophagy regulation, p62/SQSTM1 can also stabilize NFE2L2 and activate the expression of ARE genes by binding with Keap1 to block the Keap1 NFE2L2 interaction. Moreover, p62/SQSTM1 may regulate NFE2L2 in a positive feedback manner, as suggested by the fact that p62/SQSTM1 activates *NFE2L* expression by promoting autophagic degradation of Keap1 and NFE2L2 positively regulates *p62/SQSTM1* expression [100]. Taken together, these findings imply that the NFE2L2 pathway may be activated in response to oxidative stress via the autophagy-related p62/SQSTM1 pathway.

More importantly, an interaction between the p62 and mTOR pathways has been reported. In recent years, mTOR signaling has been proven to play a significant role in cell growth and metabolism [101], and it is regarded as a classical pathway to regulate autophagy [102]. mTORC1, one of the two major protein complexes of mTOR, can inhibit autophagy [102]. Raptor, which binds to mTOR in the mTORC1 complex through multiple binding regions [103], is a scaffold that binds and presents substrates to mTORC1 [104]. Deletion or knockdown of *raptor* can abolish mTORC1 activity. Interestingly, upregulation of p62 can activate mTORC1 by directly acting on raptor, a regulatory protein of mTOR, thereby suppressing autophagy [105].

Saito and colleagues showed that the NFE2L2 activator RS9 can accelerate autophagy and protect ARPE-19 cells against NaIO_3_-induced oxidative damage. ARPE-19 cells exposed to NaIO_3_ exhibited an increased LC3-II/LC-I. Notably, levels of the autophagy substrate p62/SQSTM1 were transiently elevated in the NaIO_3_ treatment group at 6 h after treatment, and the levels of LC3-I were upregulated at 24 h after NaIO_3_ treatment, implying that RS9 accelerated autophagy via transient induction of SQSTM1 expression. They also employed an intense light injury model in zebrafish to mimic in vivo AMD, and the results were in line with the in vitro experiments using ARPE-19 cells [106]. This study supports the idea that the NFE2L2 pathway plays an important role in regulating autophagy and hence preventing oxidative stress in AMD.

### 5.2. Interaction between the PGC-1 and NFE2L2 Pathways

The PGC-1 pathway, consisting of PGC-1*α*, PGC-1*β*, and PGC-1-related coactivator, serves as an antioxidant defense system targeting mitochondrial biogenesis and oxidative metabolism. AMPK and the NAD^+^-dependent deacetylase SIRT1 can activate PGC-1*α*, enhancing autophagy and mitophagy [20]. Increasing evidence supports the notion that suppression of PGC-1*α* activity contributes to the development of AMD, because loss of PGC-1*α* induces ROS generation and mitochondrial damage. In contrast, elevated expression of PGC-1*α* promotes the mitochondrial antioxidant defense by increasing the expression of antioxidant genes, such as *SOD2* and *thioredoxin 1* (*TRX1*) [107]. Thioredoxin-interacting protein can inhibit TRX activity and increase oxidative stress and destructive inflammation [108].

Zhang and colleagues generated a PGC-1*α*^+/−^ mouse model to study the pathogenesis of AMD. PGC-1*α*^+/−^ mice expressed lower levels of PGC-1*α* and were fed a high-fat diet for 4 months. The mice displayed drusen and lipofuscin accumulation, elevated ROS levels, decreased autophagy flux, and increased inflammation, along with obvious RPE and photoreceptor cell degeneration [109]. This research demonstrated that the presence of PGC-1*α* is necessary for the regulation of autophagy to prevent oxidative damage.

Felszeghy and colleagues further explored the autophagy-regulated function of both NFE2L2 and PGC-1*α* pathways in the development of dry AMD. They established and characterized a *NFE2L2/PGC-1α* double KO (dKO) mouse model to investigate the role of autophagy clearance in regulating the antioxidant response. *NFE2L2/PGC-1α* dKO mice developed severe AMD with accumulation of oxidative stress markers and damaged mitochondria. The levels of oxidative stress markers were higher than those in *NFE2L2* KO mice and *PGC-1α* KO mice, implying that *NFE2L2/PGC-1α* dKO mice exhibited the highest degree of oxidative stress. The levels of the autophagy marker p62/SQSTM1, as well as the protein aggregate-conjugated marker ubiquitin, were increased, suggesting that the UPS and autophagy clearance were impaired in *NFE2L2/PGC-1α* dKO mice. In line with the observed p62/SQSTM1 accumulation, RPE cells from dKO mice exhibited larger autolysosomes and a higher ratio of damaged mitochondria than RPE cells from WT mice, as indicated by transmission electron microscopy [40]. The study not only highlighted the significant role of intracellular degradation systems, including autophagy and the UPS, in reducing oxidative stress, but also revealed a potential crosstalk between the NFE2L2 and PGC-1*α* pathways. PGC-1*α* deficiency induces the generation of mitochondrial ROS, while the loss of NFE2L2 leads to impairment of the autophagic degradation system and the accumulation of damaged mitochondria.

### 5.3. Interaction between Autophagy and Inflammation

Autophagy may also interact with inflammation to regulate oxidative stress [110]. It is widely accepted that inflammation plays a role in the pathogenesis of AMD. Szatmari and colleagues established an in vitro AMD model by exposing human embryonic stem cell-derived RPE (hESC-RPE) cells to H_2_O_2_. Upon oxidative stress, hESC-RPE cells underwent autophagy-associated cell death. They showed that mature macrophages took up these dying cells and triggered a release of numerous proinflammatory cytokines, including interleukin- (IL-) 6, IL-8, and tumor necrosis factor-*α*, resulting in the activation of inflammatory processes. This study demonstrated that autophagy regulation may be a treatment goal to adjust inflammation and protect RPE cells from oxidative damage [14].

In conclusion, the NFE2L2 and PGC-1*α* pathways play a key role in enhancing autophagy to prevent oxidative injury. NFE2L2 upregulates autophagy by binding to AREs. AMPK activates PGC-1 and thereby promotes autophagy and mitophagy. Additionally, NFE2L2 seems to be a positive regulator of PGC-1, but the specific functional mechanisms remain unclear. Clarifying the interactions among the NFE2L2, PGC-1, AMPK, and mTOR pathways is significant to improve our understanding of the regulatory mechanisms in autophagy that alleviate oxidative stress and mitigate the development of AMD.

## 6. Potential Neuroprotective Strategies Targeting Autophagy to Alleviate Oxidative Stress in AMD

**Figure 3:**
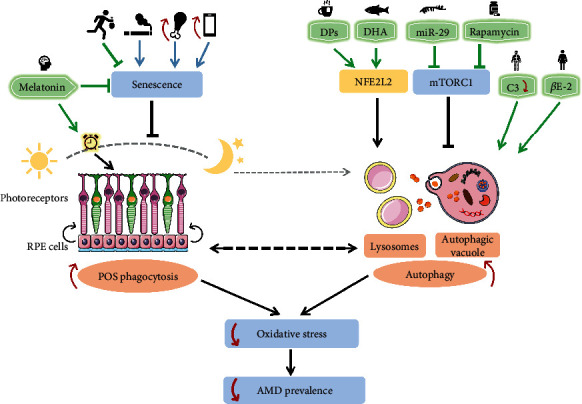
Potential neuroprotective strategies targeting autophagy to prevent oxidative damage in AMD. AMD: age-related macular degeneration; RPE: retinal pigment epithelial; POS: photoreceptor outer segments; NFE2L2: nuclear factor erythroid 2-related factor 2; mTORC1: mammalian target of rapamycin complex 1; DPs: dietary polyphenols; DHA: n-3 PUFA docosahexaenoic acid (PUFA: polyunsaturated fatty acid); miR-29: microRNA-29; C3: the third complement component; *β*E-2: 17 *β*-estradiol.

Wet, or neovascular, AMD is considered to be associated with progressive CNVs and the upregulation of VEGF [111]. Improvements in our understanding of wet AMD pathogenesis could identify and characterize therapeutic targets; for example, anti-VEGF drugs target CNV development. Unfortunately, this is the only effective AMD treatment at present, which means little advances have been made in therapies for dry AMD [17, 112]. As argued above, enhanced autophagy can mitigate oxidative stress in the pathogenesis of AMD, suggesting that stimulating autophagy may be a promising strategy for AMD therapy. Here, we summarize some neuroprotective strategies targeting autophagy to prevent oxidative damage in AMD. The overview of these neuroprotective strategies is presented in Figure 3.

### 6.1. Inhibitors for mTOR

Compelling evidence has shown that mTOR is a negative regulator of autophagy. AMPK, PI3K, and Akt perform physiological functions upstream of mTOR. The AMPK pathway inhibits mTOR activation [113], while the PI3K/Akt pathway stimulates the activation of mTOR [114]. Rapamycin is a well-known inhibitor of mTOR that has been found to increase the number of autophagic vacuoles and improve RPE and photoreceptor cell survival upon photooxidative stress [115]. Tang and colleagues also found that low doses of proteasome inhibitors, such as clasto-lactacystin-beta-lactone and epoxomicin, could increase the levels of LC3-II/LC-I and decrease the phosphorylation levels of PI3K, Akt, and mTOR in ARPE-19 cells exposed to menadione or 4-hydroxynonenal, suggesting that proteasome inhibitors can activate autophagy through blocking PI3K/Akt/mTOR signaling and prevent oxidative damage [116].

### 6.2. MicroRNAs

MicroRNAs are small endogenous RNAs that regulate the expression of genes posterior to transcription [117]. MicroRNAs have become novel therapeutic targets for various diseases due to their significant functions in response to outside influences and internal feedback [118]. Cai and Zhang discovered that overexpression of *microRNA-29* (*miR-29*) in RPE cells could rescue degenerative cells by enhancing autophagy through the inhibition of mTORC1 activity. They showed that the levels of p62 declined and LC3-II and autophagy flux increased after transfecting *miR-29* mimics into ARPE-19 cells. Moreover, protein aggregation was also repressed by knockdown of *LAMTOR1/p18*, a *miR-29* target located in the lysosome membrane [119]. Zhang and colleagues examined whether *miR-204* plays a role in regulating autophagy in RPE cells and found that knockdown of *miR-204* in both C57BL/6N mice and human RPE cells led to abnormal POS clearance and altered expression of autophagy-related proteins, indicating that high levels of *miR-204* could protect RPE cells from oxidative stress by facilitating autophagy [25]. In summary, microRNAs have proven to be effective in treating AMD, though the therapeutic mechanisms remain to be explored.

### 6.3. Hormones such as 17*β*-Estradiol and Melatonin

Wei and colleagues found that 17*β*-estradiol (*β*E-2) could enhance autophagy and protect RPE cells from blue light-emitting diode- (LED-) induced oxidative stress. After LED exposure, female ovariectomized rats, which were intravitreally injected with *β*E-2 in advance, exhibited decreased ROS levels, increased number of autophagosomes, and upregulation of p-Akt, Beclin-1, and LC3-II/LC3-I, implying that the protective mechanism of *β*E-2 is correlated with autophagy [120]. Melatonin (N-acetyl-5-methoxytryptamine) is a tryptophan-derived neurohormone that plays crucial physiological effects in many systems, for instance, the circadian rhythm, the immune system, the cardiovascular system, and the aging process [121]. Melatonin is a strong antioxidant that scavenges ROS and improves the synthesis of antioxidant enzymes [122]. It has been shown that melatonin also upregulates autophagy, protecting human RPE cells against H_2_O_2_-induced oxidative damage. Upregulation of LC3-II and Beclin-1 and downregulation of p62 have been observed after treating H_2_O_2_-exposed RPE cells with melatonin [123]. Moreover, phagocytosis of POS in higher vertebrates is synchronized with the circadian rhythms and usually occurs after dawn, suggesting that melatonin has the potential to modulate POS phagocytosis. As mentioned above, RPE cells have the ability to balance POS phagocytosis and cellular waste clearance, and increasing age can lead to dysfunction of POS phagocytosis in RPE cells. Interestingly, senescence has been reported to be associated with changes in the circadian rhythmicity of melatonin production [124]. Lysosomes, which are among the key organelles involved in autophagy, have also been found to act in a circadian rhythm-controlled manner [125]. These facts illustrate that melatonin can exert antioxidative effects by regulating autophagy.

### 6.4. Antioxidants in Diet

Several studies have revealed that some food compositions can prevent oxidative injury in AMD through regulating autophagy. Intake of dietary fish and nuts can provide marine n-3 polyunsaturated fatty acids (PUFAs) for humans [126]. Johansson and colleagues showed that physiological doses of n-3 PUFA docosahexaenoic acid (DHA), a type of PUFA, could reduce misfolded proteins and inhibit oxidative stress by enhancing autophagy through activating the NFE2L2 pathway [8]. Dietary polyphenols (DPs), which are rich in fruits, vegetables, legumes, and plant-derived beverages such as tea [127], were also found to promote autophagy by reducing impairment of the cellular waste clearance and ameliorate oxidative damage through activating the NFE2L2 pathway, thereby preventing the development of AMD [128].

### 6.5. Complement Depletion

The complement system is widely believed to be responsible for regulating the immune system and inflammation [129]. Complement depletion has been found to improve autophagic activity, reduce cellular oxidative stress, and mitigate age-related retinal degeneration. McHarg and colleagues investigated the role of the third complement component (C3) in AMD and found that *C3* transcription is upregulated in aged retinas [130]. They also evaluated the thickness of retinas in C3-deficient mice through spectral domain optical coherence tomography and showed that the retinas of C3-deficient mice aged 12 months were thinner than those of WT mice aged 3 months, implying that complement activation plays a role in the natural process of retinal aging. Additionally, LC3-II/LC-I in C3-deficient mice was higher than that in WT mice [131]. These findings indicated that C3 is associated with autophagy regulation and may be a promising therapeutic target for AMD.

## 7. Conclusions

Many risk factors contribute to the development of AMD, including light injury, growing age, and cigarette smoke (as shown in Figure 1). They can aggravate ROS production and thus trigger excessive cellular oxidative stress, causing disorders of the cell structure and function. Enhanced autophagy can alleviate oxidative damage in AMD and protect RPE and photoreceptor cells from degeneration and death. Remarkably, overactivated autophagy may also lead to cell death at the early stages of retinal degenerative diseases. Thus, defining the precise dynamic role of autophagy in the pathogenesis of AMD is essential to choose optimal time points for neuroprotection. As illustrated in Figure 2, the crosstalk among the NFE2L2, PGC-1, p62, AMPK, and PI3K/Akt/mTOR pathways may play a crucial role in enhancing autophagy to prevent oxidative injury. Recently, some novel neuroprotective strategies (as shown in Figure 3) targeting these signaling pathways to activate autophagy and improve RPE and photoreceptor cell survival have been described. However, further studies are still needed to elucidate the precise interaction among these pathways in order to provide more therapeutic interventions, considering that currently there are no effective treatments for dry AMD.
